# Author Correction: Ribosomal modifications are associated with mesenchymal fate selection in the neural crest lineage

**DOI:** 10.1038/s41467-026-77321-6

**Published:** 2026-08-27

**Authors:** Irina Poverennaya, Aliia Murtazina, Lei Li, Lorena Maili, Lukas Sourada, Luis Fernando Montano-Gutierrez, Rozalina Galimullina, Tobias Steinschaden, Marketa Kaiser, Tomas Zikmund, Adna Goralija, Teng Gao, Aurore Attina, Ornella Clara, Christoph Bartenhagen, Alek G. Erickson, Yaakov Gershtein, Shiyuan Chen, Kristyna Polaskova, Jaroslav Sterba, Bettina Semsch, Emma R. Andersson, Varsha Prakash, Theresa Vincent, Maria Arceo, Per Kogner, Susanne Schlisio, Peter V. Kharchenko, Alexandre David, Jozef Kaiser, Matthias Fischer, Jan Skoda, Paul A. Trainor, Andrei S. Chagin, Igor Adameyko

**Affiliations:** 1 Department of Neuroimmunology, Center for Brain Research, Medical University of Vienna, Vienna, Austria; 2 Department of Physiology and Pharmacology, Karolinska Institutet, Stockholm, Sweden; 3 Department of Internal Medicine and Clinical Nutrition, Institute of Medicine, Centre for Bone and Arthritis Research at the Sahlgrenska Academy, Gothenburg University, Gothenburg, Sweden; 4 Stowers Institute for Medical Research, Kansas City, MO USA; 5 Department of Experimental Biology, Faculty of Science, Masaryk University, Brno, Czech Republic; 6 International Clinical Research Center, St. Anne’s University Hospital, Brno, Czech Republic; 7 Central European Institute of Technology, Brno University of Technology, Brno, Czech Republic; 8 Department of Biomedical Informatics, Harvard Medical School, Boston, MA USA; 9 IRCM, Université de Montpellier, ICM INSERM, Montpellier, France; 10 Aix-Marseille University, CNRS, UMR 7288, IBDM, Marseille, France; 11 Department of Experimental Pediatric Oncology, University Children’s Hospital of Cologne, Cologne, Germany; 12 Center for Molecular Medicine Cologne, Medical Faculty, University of Cologne, Cologne, Germany; 13 Department of Molecular Biosciences, the Wenner-Gren Institute, Stockholm University, Stockholm, Sweden; 14 Department of Pediatric Oncology, University Hospital Brno and Faculty of Medicine, Masaryk University, Brno, Czech Republic; 15 Department of Cell and Molecular Biology, Karolinska Institutet, Stockholm, Sweden; 16 Department of Microbiology, NYU Grossman School of Medicine, New York, NY USA; 17 Division of Pathology, Department of Laboratory Medicine, Karolinska Institutet, Stockholm, Sweden; 18 Department of Oncology-Pathology, Karolinska Institutet, Stockholm, Sweden; 19 Childhood Cancer Research Unit, Department of Women’s and Children’s Health, Karolinska Institutet, Stockholm, Sweden; 20 Altos Labs, San Diego Institute of Science, San Diego, CA USA; 21 IRMB-PPC, INM, CHU Montpellier, INSERM, Université de Montpellier, CNRS, Montpellier, France; 22 Department of Cell Biology and Physiology, University of Kansas Medical Center, Kansas City, KS USA; 23 Science for Life Laboratory, Institute of Medicine, University of Gothenburg, Gothenburg, Sweden; 24 Present Address: Division of Hematology/Oncology, Boston Children’s Hospital, Boston, MA USA

**Keywords:** Differentiation, Gene regulatory networks, Cell lineage, CNS cancer

Correction to: *Nature Communications* https://doi.org/10.1038/s41467-026-70375-6, published online 9 March 2026

In the version of this article initially published, the surname of Bettina Semsch was misspelled (Semasch), the surname of Emma R. Andersson was misspelled (Anderson), and the middle name initial of Alek G. Erickson was missing. The names have been amended in the HTML and PDF versions of the article.

